# Could carbonic anhydrase IX predict fetal growth restriction in early onset preeclampsia?

**DOI:** 10.1590/1806-9282.20250838

**Published:** 2025-12-08

**Authors:** Gulten Ozgen, Burcu Dincgez

**Affiliations:** 1 University of Health Sciences, Bursa Yuksek Ihtisas Training and Research Hospital, Department of Obstetrics and Gynecology – Bursa, Turkey.

**Keywords:** Carbonic anhydrase IX, Fetal growth restriction, Preeclampsia

## Abstract

**OBJECTIVE:**

Placental insufficiency, which plays a crucial role in both preeclampsia and fetal growth restriction, can cause oxidative stress and high reactive oxygen species production that could lead to hypoxia. Carbonic anhydrase IX is induced by hypoxia-inducible factor-1α, an important transcriptional regulator of genes involved in response to hypoxia. Considering this, we aimed to determine carbonic anhydrase IX levels in early preeclampsia with and without fetal growth restriction, and also to evaluate the predictive role of carbonic anhydrase IX for the development of fetal growth restriction in early onset preeclampsia.

**METHODS:**

This prospective study included a total of 180 participants, who were divided into two groups—early onset preeclampsia (n=90) and control (n=90). Then, preeclamptic patients were followed and subgrouped as preeclampsia with fetal growth restriction (n=35) and without fetal growth restriction (n=55). Sociodemographic and obstetric characteristics and laboratory features including carbonic anhydrase IX were compared between groups.

**RESULTS:**

Median carbonic anhydrase IX was higher in preeclampsia than controls [2.07 (0.78–5.48) vs. 1.32 (0.63–2.12), p<0.001]. Moreover, median carbonic anhydrase IX was higher in the preeclampsia with fetal growth restriction group as compared to the preeclampsia without fetal growth restriction group [2.76 (0.78–5.48) vs. 1.77 (0.89–5.14), p=0.018]. The carbonic anhydrase IX >1.83 pg/mL predicted fetal growth restriction with 77.14% sensitivity and 52.73% specificity (p=0.015, AUC=0.649) in the early onset preeclampsia.

**CONCLUSION:**

Carbonic anhydrase IX could reflect the insufficiency of the placenta in early onset preeclampsia and fetal growth restriction, and it could be used in early onset preeclampsia patients for the prediction of fetal growth restriction.

## INTRODUCTION

Preeclampsia is one of the most common pregnancy complications with a complex etiology that greatly affects perinatal morbidity and mortality^1^. Placental insufficiency due to various placental pathologies, such as maternal and fetal vascular malperfusion, constitutes the common etiology of this complex syndrome^2^. Placental malperfusion and vascular damage can cause oxidative stress, inflammation, and high reactive oxygen species production that could lead to hypoxia^3^. Fetal growth restriction (FGR) is recognized to be linked to hypoxia pathways in preeclampsia, especially early onset^4^. It complicates nearly 30% of preeclampsia cases^5^. Due to the increased adverse perinatal outcomes, many studies have focused on searching for biomarkers related to placental insufficiency, oxidative stress, and hypoxia in FGR and preeclampsia to provide appropriate management. Increased sFlt-1/PlGF ratio for placental insufficiency has been accepted as a classical biomarker indicating preeclampsia and/or FGR. Additionally, interleukin (IL)-1 and IL-6 have been found to be associated with hypoxia-induced oxidative stress in the FGR^6^.

Carbonic anhydrase IX (CAIX) is a member of the zinc-containing carbonic anhydrase family, which consists of 16 isoenzymes^7^. It is a transmembrane protein that maintains cellular pH and supports cell survival under hypoxic conditions^8^. Carbonic anhydrase IX is induced by hypoxia-inducible factor-1α (HIF-1α), an important transcriptional regulator of genes involved in response to hypoxia, and its release is upregulated in various cancers, including renal cell cancer. This induction is a major metabolic effector of tumor hypoxia, as CAIX catalyzes the metabolism of CO_2_ to carbonic acid and is associated with poor prognosis in various tumors such as breast cancer^9,10^.

There is little knowledge about the relationship between preeclampsia and CAIX. In a study, CAIX was claimed to be an early predictor of preeclampsia, while other studies demonstrated increased levels of CAIX in preeclampsia and HELLP syndrome^11-13^.

To the best of our knowledge, there is no study in the ­literature searching for the relationship between CAIX and FGR in early onset preeclampsia (EOP). In the present study, we aimed to determine CAIX levels in the EOP with and without FGR and also to evaluate the predictive role of CAIX for FGR in the EOP.

## METHODS

This prospective study is conducted at Bursa Yuksek Ihtisas Research and Training Hospital between November 2023 and January 2025. The local ethics committee approved the study. All procedures in the study were performed in accordance with the Helsinki Declaration. Written informed consent was taken from all study participants.

### Study population

A total of 212 pregnant women with hypertensive disorders of pregnancy after 20 weeks of gestation were enrolled. The study's inclusion criteria included being between 18 and 45 years old, having a diagnosis of EOP, receiving regular prenatal care, delivering at our hospital, and giving informed consent to participate in the study. Patients with multiple pregnancies, fetal congenital anomalies, diabetes, premature rupture of membranes, renal or liver diseases, recurrent miscarriage, deep vein thrombosis, antiphospholipid syndrome, malignancies, thrombophilias, other hypertensive disorders of pregnancy except preeclampsia, HELLP syndrome, smokers, and taking nonsteroidal anti-inflammatory, anticoagulant, and antihypertensive drugs were excluded from the study. A total of 118 pregnant women with EOP were included as a patient group. In antenatal follow-up, 2 patients were complicated with HELLP syndrome, 11 with premature rupture of membranes, 6 with gestational diabetes. A total of 9 patients were withdrawn from the study. Consequently, 90 patients with EOP were analyzed for the study.

Preeclampsia was diagnosed according to the American College of Obstetricians and Gynecologists guideline as follows: new-onset hypertension after 20 weeks of gestation accompanied by proteinuria or end organ damage such as thrombocytopenia, elevated transaminases, increased creatinine, pulmonary edema, and/or visual symptoms in the absence of proteinuria. EOP was defined as the occurrence of preeclampsia before 34 weeks of gestation^14^.

For the control group, pregnant women aged 18–45 with no pregnancy complications or known diseases, who received regular prenatal care, delivered at our hospital, and provided informed consent were included. A total of 113 age- and gestational week–matched pregnant women participated in the study. In the follow-up, 8 patients were complicated with premature rupture of membranes and 7 with gestational diabetes. A total of 5 patients gave birth at a different obstetrics clinic and 3 of them were withdrawn. Finally, we analyzed 90 patients for the control group. Preeclamptic patients were followed and subgrouped as EOP with FGR (n=35) and EOP without FGR (n=55).

FGR was defined according to the Delphi procedure. These procedures were as follows: 1. Abdominal circumference <3rd centile or estimated fetal weight <3rd centile or absent end diastolic flow in the umbilical artery, or 2. Both of the following: estimated fetal weight or abdominal circumference <10th centile and pulsatility index of the uterine artery >95th centile or pulsatility index in the umbilical artery >95th centile^15^. The percentiles were calculated according to The Fetal Medicine Foundation calculation application^16^.

Age, body mass index (BMI), parity, gestational week when the samples were taken, blood pressure, delivery week and mode, birth weight, baby gender, 1st and 5th minute Apgar scores, neonatal intensive care unit admission, platelet count, transaminase and creatinine levels, and development of FGR were recorded.

### Biochemical analysis

For late second trimester serum CAIX measurements, serum samples were obtained at admission. A total of 325 blood samples (212 hypertensive disorders of pregnancy and 113 control) were collected. The 10 mL blood samples were centrifuged at 1,800 rpm for 10 min and stored at −80 degrees centigrade. In the antenatal follow-up, patients were grouped as EOP and control. Then, serum samples were analyzed for only 180 participants. Maternal CAIX was measured using an enzyme-linked immunosorbent assay method with a commercial kit (Human CA9 Elisa Kit, A.B.T™. Laboratory Industry, Cat: ABT1007Hu, Ankara, Turkey). The measurement range was 7.8–500 pg/mL, and the coefficient of variability is 3.8% (intra-assay) and 6.3% (inter-assay).

### Statistical analysis

The Shapiro-Wilk test was used to assess the assumption of normality. While the student t-test was used to compare normally distributed quantitative data between two groups, Mann-Whitney U test was used to evaluate non-normally distributed quantitative data. Categorical data were compared using the chi-square or Fisher's exact test. The mean±standard deviation, median (minimum–maximum), and frequency (related ­percentage) were used to represent descriptive statistics. To determine the predictive role of CAIX for FGR in the EOP, ROC ­analysis was performed. The sensitivity, specificity, Youden index, and cut-off values were analyzed. The statistical analyses were performed using the MedCalc18 and SPSS 22.0 programs, and the significance level was considered as α=0.05.

## RESULTS

The sociodemographic, clinical, and perinatal characteristics of the EOP and control groups are demonstrated in Table 1. No significant difference was detected between the EOP and control groups regarding age, BMI, gravida, parity, nulliparity rate, gestational age at sampling, and the rates of neonatal death. Systolic and diastolic blood pressure, the rates of preterm birth, cesarean section, and NICU requirement were significantly higher, whereas birth weight, birth week, and Apgar scores at the first and fifth minutes were lower in the EOP group as compared to the control group.

**Table 1:** The sociodemographic, clinical, perinatal, and laboratory characteristics of early onset preeclampsia and control groups.

	EOP (n=90)	Control (n=90)	p-value
Age (years)	27 (18–40)	27.5 (18–40)	0.444
BMI (kg/m^2^)	31.4 (23.8–48.3)	33 (21.3–46.9)	0.119
Gravida (n)	2 (1–7)	3 (1–6)	0.490
Parity (n)	2 (0–7)	2 (0–6)	0.637
Nulliparity (n, %)	3 (3.3%)	6 (6.7%)	0.497
Gestational age at sampling (week)	24 (22–26)	24 (22–26)	0.986
Systolic blood pressure (mmHg)	140 (140–200)	120 (110–130)	**<0.001**
Diastolic blood pressure (mmHg)	90 (90–110)	80 (60–90)	**<0.001**
Birth weight (gram)	1,955 (525–4,780)	3,305 (845–4,200)	**<0.001**
Birth week (week)	35 (24–39)	38 (28–41)	**<0.001**
Apgar score of first minute	7 (1–9)	8 (2–9)	**<0.001**
Apgar score of fifth minute	8 (2–10)	9 (1–10)	**0.034**
NICU requirement (n, %)	18 (20%)	8 (8.9%)	**<0.001**
Preterm birth (n, %)	61 (67.8%)	19 (21.1%)	**<0.001**
Cesarean section (n, %)	40 (44.4%)	17 (18.9%)	**<0.001**
Neonatal death (n, %)	5 (5.6%)	3 (3.3%)	0.469
Platelet (x10^3^/μL)	211 (109–417)	202.5 (125–361)	0.233
AST (IU/L)	21.5 (10–57)	19.5 (7–59)	0.062
ALT (IU/L)	12.5 (2–36)	12 (6–54)	0.439
Creatinine (mg/dL)	0.58 (0.43–1.40)	0.47 (0.4–0.87)	0.175
Maternal CAIX (pg/mL)	2.07 (0.78–5.48)	1.32 (0.63–2.12)	**<0.001**

ALT: alanine aminotransferase; AST: aspartate aminotransferase; BMI: body mass index; CAIX: carbonic anhydrase IX; EOP: early onset preeclampsia; NICU: neonatal intensive care unit. Mann-Whitney-U test was used to compare variables between two groups for continuous variables and expressed as median (min–max) values. Significant p-values were expressed in bold.

The laboratory characteristics of the EOP and control groups are shown in Table 1. Platelet count, aminotransferases, and creatinine levels did not differ between the two groups. Median maternal CAIX was 2.07 (0.78–5.48) pg/mL in the EOP group and 1.32 (0.63–2.12) pg/mL in the control group, which was statistically higher in the EOP group (p<0.001). Table 2 summarizes the sociodemographic, clinical, perinatal, and laboratory characteristics of the EOP with and without FGR. Statistical analysis revealed that there was no difference between the two groups in age, BMI, gravida, parity, nulliparity rate, gestational age at sampling, systolic and diastolic blood pressure, and the rates of neonatal death. In the EOP with the FGR group, birth weight was lower (p<0.001), birth week was earlier (p=0.011), Apgar scores of the first and fifth minutes were lower (p<0.001 for both), NICU requirement was higher (p=0.031), and preterm birth rate was higher (p=0.015) than in the EOP without the FGR group. The laboratory characteristics of EOP with and without FGR groups are presented in Table 2. No significant difference was detected in terms of platelet count, aminotransferases, and creatinine levels. Median maternal CAIX was 2.76 (0.78–5.48) pg/mL in the EOP with the FGR group and 1.77 (0.89–5.14) pg/mL in the EOP ­without the FGR group, which was statistically higher in the EOP with the FGR group (p=0.018).

**Table 2:** The sociodemographic, clinical, perinatal, and laboratory characteristics of early onset preeclampsia with fetal growth restriction and early onset preeclampsia without fetal growth restriction cases.

	EOP with FGR (n=35)	EOP without FGR (n=55)	p-value
Age (years)	26 (19–39)	27 (18–40)	0.054
BMI (kg/m^2^)	30.9 (23.8–48.3)	33 (24–48.3)	0.410
Gravida (n)	2 (1–7)	2 (1–7)	0.487
Parity (n)	2 (0–7)	2 (0–5)	0.756
Nulliparity (n, %)	1 (2.9%)	2 (3.6%)	1.000
Gestational age at sampling (week)	24 (22–26)	24 (22–26)	0.616
Systolic blood pressure (mmHg)	150 (140–180)	140 (140–200)	0.192
Diastolic blood pressure (mmHg)	90 (90–110)	90 (90–110)	0.795
Birth weight (gram)	1,547±543.7	2,360±821.5	**<0.001** *
Birth week (week)	34 (24–38)	36 (25–39)	**0.011**
Apgar score of first minute	6 (1–9)	8 (3–9)	**<0.001**
Apgar of score fifth minute	7 (2–10)	9 (4–10)	**<0.001**
NICU requirement (n, %)	11 (31.4%)	7 (12.7%)	**0.031**
Preterm birth (n, %)	29 (82.9%)	32 (58.2%)	**0.015**
Cesarean section (n, %)	20 (57.1%)	20 (36.4%)	**<0.001**
Neonatal death (n, %)	4 (11.4%)	1 (1.8%)	0.052
Platelet (x10^3^/μL)	220 (132–323)	206 (109–417)	0.665
AST (IU/L)	23 (12–42)	21 (10–57)	0.526
ALT (IU/L)	14 (6–36)	12 (2–28)	0.169
Creatinine (mg/dL)	0.58 (0.44–1.36)	0.58 (0.43–1.40)	0.960
Maternal CAIX (pg/mL)	2.76 (0.78–5.48)	1.77 (0.89–5.14)	**0.018**

ALT: alanine aminotransferase; AST: aspartate aminotransferase; BMI: body mass index; CAIX: carbonic anhydrase IX; EOP: early onset preeclampsia; NICU: neonatal intensive care unit; FGR: fetal growth restriction. Mann-Whitney U test was used to compare variables between two groups for continuous variables and expressed as median (min–max) values.

* Student t-test was used to compare normally distributed continuous variables between two groups and expressed as mean±SD. Significant p-values were expressed in bold.

**Figure 1:**
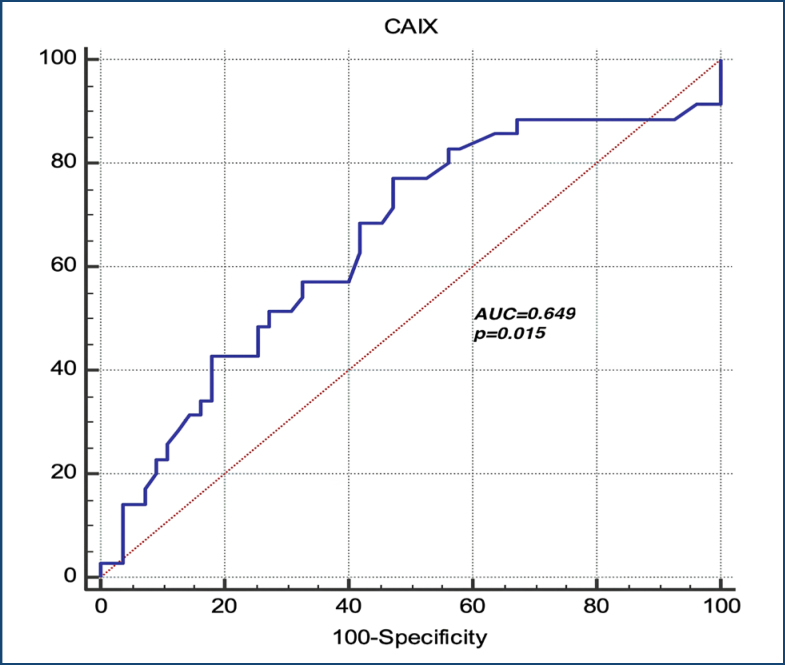
The ROC curve evaluating the role of carbonic anhydrase IX in predicting fetal growth restriction in preeclampsia.

The ROC curve evaluating the role of CAIX in predicting FGR was demonstrated in Figure 1. According to this analysis, CAIX >1.83 pg/mL predicted the FGR with 77.14% sensitivity and 52.73% specificity (p=0.015, AUC=0.649) in the EOP.

## DISCUSSION

Placental insufficiency, the pathophysiology of which is not fully elucidated, plays an important role both in preeclampsia and FGR. The imbalance in inflammatory, anti-angiogenic, and hypoxia-related factors could lead to placental insufficiency^17,18^. The recent approach has focused on identifying markers that detect placental insufficiency^19^. Unfortunately, there is no marker representing early findings of placental insufficiency in current practice. In a study by Schoots et al., the discriminative role of free thiols, ischemia-modified albumin, leptin, and soluble receptors for advanced glycation end products has been assessed in FGR and preeclampsia. Although none of the biomarkers have discriminated FGR from healthy controls, all of them discriminated FGR+PE from healthy controls and FGR only^20^.

Hypoxia-inducible factor-1α (HIF-1α), CAIX, and microRNAs have been claimed to be involved in hypoxia-related processes^17^. Hypoxia-inducible factor-1α is regulated by the oxygen levels of the tissue. It degrades in normoxia while it accumulates in related hypoxic conditions^17,21^. Carbonic anhydrase IX, induced by HIF-1α, is another transmembrane protein related to hypoxia^9^. It has been studied as a potential marker for predicting preeclampsia and HELLP syndrome^11,13^. In a study by Galbiati et al., it was reported that CAIX levels significantly increased in women who later developed preeclampsia, and CAIX had a better predictive role than the sFlt1/PIGF ratio for preeclampsia^11^. In another study, third trimester CAIX levels were found to be elevated in preeclampsia as compared to normal pregnancies^12^. Similar to those studies, Mentese et al. reported higher CAIX levels in HELLP syndrome, which is a severe form of preeclampsia^13^. Consistent with the literature, the present study found significantly higher late second trimester CAIX levels in the EOP patients as compared to normal pregnancies. The results of all these studies reflect that hypoxia-induced elevated CAIX plays a crucial role in the pathophysiology of preeclampsia. Although EOP accounts for one-fifth of preeclampsia cases, it is responsible for almost 80% of preeclampsia-related complications. Considering that finding markers in EOP is more important than finding markers in late onset preeclampsia^22^.

Different from those studies, the present study categorized preeclamptic patients based on whether or not FGR was ­present and higher maternal serum CAIX in preeclamptic patients who developed FGR. Moreover, CAIX>1.83 pg/mL predicted FGR with 77.14% sensitivity and 52.73% specificity in EOP patients. These findings can indicate the role of CAIX in detecting the severity of placental insufficiency.

Doppler velocity measurements can aid in determining impaired placental functions in the FGR cases. While uterine artery Doppler reflects the resistance in maternal circulation, umbilical artery Doppler shows the placental circulation resistance^23^. In early FGR, a high umbilical artery pulsatility index can indicate placental insufficiency^24^. Then, absent or reversed end diastolic flow occurs. After 2 or 3 weeks from this deterioration, impaired fetal well-being could be detected in cardiotocography^23^. Considering that umbilical artery Doppler abnormalities develop when more than half of the placenta is dysfunctional, early markers predicting placental dysfunction in FGR are of great importance. We suggest that higher CAIX levels could be a representative marker for placental insufficiency in FGR cases. Further studies are needed to clarify the relationship between CAIX levels, Doppler, and pathological placental findings in the EOP patients complicated with the FGR.

Data arising from a single center with a limited case number was the main limitation of the present study. However, this is the first study searching for the role of CAIX in the EOP and FGR.

## CONCLUSION

In conclusion, we suggest that CAIX could reflect the insufficiency of the placenta in the EOP and FGR, and it could be used in EOP for the prediction of the FGR.

## ETHICAL APPROVAL

This study was approved by the local ethics committee (Number: 2024-TBEK; Date: 2024/10-15).

## Data Availability

The datasets generated and/or analyzed during the current study are available from the corresponding author upon reasonable request.
